# The challenges and benefits of public health in smart cities from a 4 M perspective

**DOI:** 10.3389/fpubh.2024.1361205

**Published:** 2024-06-03

**Authors:** Lirong Yuan, Lihong Du, Yonggang Gao, Yujin Zhang, Yongqing Shen

**Affiliations:** ^1^Hebei University of Chinese Medicine, Shijiazhuang, China; ^2^Traditional Chinese Medicine Health Care Research Key Laboratory Project of Hebei Province, Shijiazhuang, China

**Keywords:** 4 M perspective, smart city, public health, depth of benefits, neural network

## Abstract

**Introduction:**

With the acceleration of urbanization, public health issues have become increasingly prominent in smart city construction, especially in the face of sudden public health crises. A deep research method for public health management based on a 4M perspective (human, machine, materials, methods) is proposed to effectively address these challenges. Methods: The method involves studying the impact of human factors such as population age, gender, and occupation on public health from a human perspective. It incorporates a machine perspective by constructing a public health prediction model using deep neural networks. Additionally, it analyzes resource allocation and process optimization in public health management from the materials and methods perspectives.

**Results:**

The experiments demonstrate that the public health prediction model based on deep neural networks achieved a prediction accuracy of 98.6% and a recall rate of 97.5% on the test dataset. In terms of resource allocation and process optimization, reasonable adjustments and optimizations increased the coverage of public health services by 20% and decreased the response time to public health events by 30%.

**Discussion:**

This research method has significant benefits for addressing the challenges of public health in smart cities. It can improve the efficiency and effectiveness of public health services, helping smart cities respond more quickly and accurately to potential large-scale public health events in the future. This approach holds important theoretical and practical significance.

## 1 Introduction

With the acceleration of urbanization, urban development also faces many challenges, one of which is public health (PH) issues (1, 2). With the development of technology, the concept of smart cities is gradually being proposed. Its goal is to utilize advanced information technology to enhance the operational efficiency of cities, improve the quality of life of citizens, and effectively address various urban issues (3, 4). However, in the process of building smart cities, the importance of PH issues has become increasingly prominent, especially in the face of sudden PH crises. PH issues involve multiple aspects such as urban pollution, food safety, disease control, etc. These issues not only directly affect the quality of life of citizens, but also have a profound impact on the long-term development of cities. In addressing these issues, this study requires more effective tools and methods. Previous methods often relied on traditional data collection and processing methods, but these methods may appear inadequate when faced with large-scale data and complex problems. Therefore, how to utilize new technologies and methods to optimize PH management has been an urgent issue to be addressed. Conducting in-depth research on PH management from the perspective of 4 M (human, machine, material, method) will help to better understand and solve this problem. The integration of the 4 M perspective and DNN technology promotes innovation in public health management through a comprehensive analysis of human behavior, technological potential, infrastructure needs, and system procedures. DNN can analyze large datasets to provide information for health strategies, and this integration can enable more comprehensive resource allocation for intervention measures and health solutions. In terms of methodology, this study first starts from a human perspective and investigates the impact of factors such as population age, gender, and occupation on PH. It further introduces a machine perspective and constructs a PH prediction model on the ground of deep neural networks (DNN). Finally, a systematic analysis of resource allocation and process optimization in PH management will be conducted from the perspectives of materials and methods. The innovation lies in the first introduction of the concept of 4 M into PH management, combined with DNN technology. This provides new theories and tools for predicting and managing PH. In addition, research on resource allocation and process optimization also provides new perspectives and methods for the practice of PH management. For the PH management of smart cities, research methods have important theoretical and practical significance. It can not only improve the efficiency and effectiveness of PH services, but also help smart cities respond more quickly and accurately to potential large-scale PH events (5, 6). Implementing the proposed research plan is expected to enhance public health services in smart cities by offering personalized healthcare interventions, predicting outbreaks using data analytics, and optimizing resource allocation. For large-scale public health events, this approach could enable rapid, data-driven responses, ensuring timely and targeted measures are taken to mitigate risks and manage situations effectively, thereby improving overall public health resilience. It is expected that the research plan can play a positive role in promoting PH and provide new theoretical and practical references for PH management in smart cities. It also hopes to inspire more researchers to explore PH issues from different perspectives and levels. This will make greater contributions to the construction of smart cities and the improvement of PH services. The study will explore how to enhance the public health management of smart cities through the 4 M framework and deep neural network technology, assuming that this integrated approach can improve the efficiency and effectiveness of responding to public health crises. The research will be conducted in four parts. The first is an overview of the challenges and benefits of PH in smart cities from a 4 M perspective. The second is a model of the challenges and benefits of PH in smart cities on the ground of a 4 M perspective. The third is the relevant verification of the second. The fourth is a summary of the research content and points out the demerits.

## 2 Literature review

With the continuous acceleration of urbanization, the importance of Public Health (PH) issues in the construction of smart cities is becoming increasingly prominent. van Hoogstraten et al. (7) combined with urinary biomarkers, are effective in the diagnosis of bladder cancer, which is crucial to cope with the shortage of professionals and limited resources. It is also crucial to improve the whole society’s understanding of the risks and symptoms of bladder cancer, and to study the relationship between lifestyle and the prognosis of bladder cancer. The impact and challenges of current epidemiological trends on PH and clinical practice deserve in-depth research (7). Garcia Casal et al. proposed accurate and economically affordable diagnostic tools for anemia, which can help understand the scale and distribution of diseases and develop prevention and treatment measures. Hemoglobin testing is the main diagnostic method for anemia, and venous blood and automated hematology analyzers are preferred. The consequences of misdiagnosis highlight the importance of accurate diagnosis (8). Falahee B et al. conducted a randomized controlled trial using patient-centered personalized management Individualized Management for Patient-Centered Targets (IMPaCT). This experiment is a standardized intervention for community health workers aimed at addressing the unmet social needs of vulnerable groups. The analysis results indicate that for every dollar invested in IMPaCT, an average return of $2.47 will be brought to healthcare recipients during the fiscal year (9). Mitchell et al. proposed a non vaccinated disease avoidance plan for overseas immigrants on the ground of health assessments. The Centers for Disease Control and Prevention and the US Department of State jointly developed and funded a global immunization program for refugees traveling to the United States. This plan was implemented in collaboration with the International Organization for Migration in 2012 (10). Munshi H et al. proposed feasible alternative solutions to address the gaps and challenges faced in global health. The origin of the term global health can be traced back to the outdated term tropical medicine. The asymmetry of power dynamics between high-income countries and low - to middle-income countries is a core issue in today’s global health structure (11).

To more effectively address these challenges, in-depth research on PH management on the ground of the 4 M perspective is gradually receiving attention. Zhu S and others are introducing zero emission technology to replace traditional freight systems in California to address air quality issues in communities near major ports. Among them, hydrogen fuel cell powered equipment and vehicles are considered as zero emission solutions for port freight technology. The research results indicate that the comprehensive use of Fuel Cell Electric Equipment and Technology (FCEET) can significantly improve the levels of maximum 8-h ozone and 24-h PM2.5. And its estimated health benefits range from 3.21 million to 7.11 million US dollars per day (12). Net P et al. analyzed the recommended influenza vaccines since 2010 and evaluated the impact of high-dose Trivalent Influenza Vaccine (TIV) by establishing a budget impact model and decision tree framework. The research results indicate that over a period of 10 years, high-dose TIV has avoided over 1.33 million influenza cases, saved $4.6 billion, and converted into a return on investment of 21.44% (13). Hojsak et al. proposed that fiber is an essential nutrient in the diet. It provides a variety of health benefits, such as fecal distension, cholesterol reduction, control of blood sugar and weight, as well as systemic health benefits from the fermentation of fiber by the gut microbiota. Its deficiency is related to children’s constipation, irritable bowel syndrome, allergies, and immune related diseases. In the research results, the understanding of fibers should be on the ground of physiological functions rather than physical and chemical properties, and fiber intake in healthy children and children with functional gastrointestinal diseases should be quality oriented. Meanwhile, the importance of gut microbiota needs to be considered (14). Giubilini et al. proposed ethical standards to determine whether the task of vaccinating healthcare workers is morally reasonable. These standards include the efficacy of vaccines for healthcare workers and patients, and the existence of alternative solutions that can achieve the same benefits. The research results indicate that when vaccination on the ground of these standards is reasonable, they are not unfair discrimination, and the degree of coercion involved is morally acceptable. Even if the vaccination task for the general population is unreasonable, the vaccination task for healthcare workers may still be reasonable (15). Recent research by Worrell et al. suggests that state restrictions or complete bans on abortion are putting women’s psychological and medical health at risk. These restrictions may prevent many women from accessing safe abortion services, thereby increasing their likelihood of facing psychological problems and medical risks. Psychological research has been ongoing for 50 years. These studies reveal the harmful effects of refusing abortion and provide valuable insights for PH leaders on how to facilitate women’s reproductive rights and maternal and child health. These studies provide ample evidence that abortion is not related to mental health issues. The opposite statement is incorrect because these studies are on the ground of extensive scientific evidence and expert consensus. These studies also provide valuable recommendations on how to protect women’s rights and improve their healthcare. Therefore, these research findings should be carefully considered and measures taken to protect women’s rights and health (16).

The research on PH in smart cities on the ground of the 4 M perspective provides a new perspective and theoretical tool for understanding and solving PH problems. This effectively promotes theoretical innovation and practical development in the field of PH. There are still some areas for improvement in the research, such as from a human perspective, more in-depth subgroup studies will contribute to more precise PH management. In the machine perspective, further optimization of Deep Neural Networks (DNNs) will improve the accuracy of predictions. In addition, further research from the perspectives of materials and methods is needed to achieve more efficient PH management. In the future, it is expected that research methods on the ground of the 4 M perspective can be promoted and applied globally, contributing to the PH management of smart cities.

## 3 A deep model of PH challenges and benefits in smart cities from a 4 M perspective

It establishes a smart city PH benefit factor evaluation system, providing a systematic framework for measuring and evaluating PH benefits. It constructs a 4 M smart city PH DNN model, comprehensively analyzing the mechanism of PH management process from four perspectives: human, machine, material, and method. Finally, on the ground of DNNs, a deep model of PH benefits in smart cities from a 4 M perspective is proposed, providing a new tool for predicting and managing PH. The proposed research methodology for tackling public health challenges in smart cities integrates human factors, technology, infrastructure, and procedural strategies. It focuses on how behaviors and demographics interact with advanced technologies like deep neural networks for better health outcomes. The methodology also incorporates the role of healthcare infrastructure and environmental elements, along with the implementation of systematic public health policies and protocols in urban settings.

### 3.1 Establishment of a smart city PH benefit factor evaluation system

**Figure 1:**
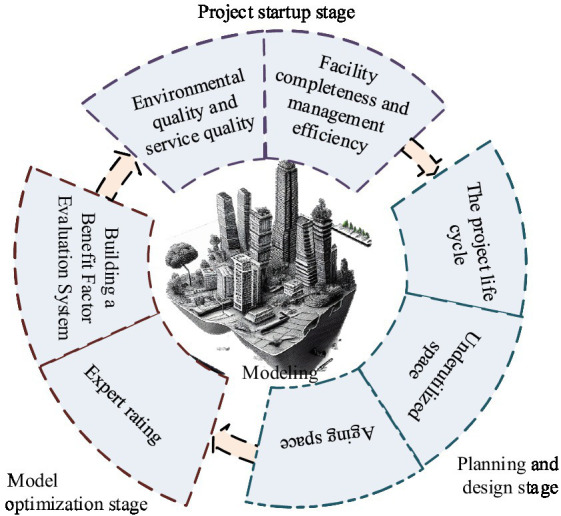
Evaluation of PH benefit factors in smart cities.

When establishing a deep model of PH benefits in smart cities on the ground of the 4 M perspective, the primary task is to construct a PH benefit evaluation system. This involves a systematic classification of PH efficiency factors, including environmental quality, service quality, facility completeness, management efficiency, etc. PH space is divided into surplus space and negative space. Surplus space is a space that has not been fully utilized during the planning and design stage, while negative space is an aging space due to poor management or insufficient functionality. Improving these spaces can refer to project lifecycle theory and optimize from multiple stages. Through this evaluation system, this study can conduct in-depth analysis and optimization of PH management in smart cities, further enhancing PH benefits (17, 18). The implementation of practical work should be on the ground of the lifecycle theory of the project, and should be carried out from multiple stages. The evaluation of PH benefits factors for smart cities is shown in Figure 1.

In establishing a deep model of PH benefits in smart cities on the ground of the 4 M perspective, select appropriate PH benefits factors and obtain relevant data, and quantitatively evaluate the factors through data analysis and model establishment. After the completion of the model, a comprehensive acceptance is conducted to ensure its accuracy and effectiveness. After running the model for a period of time, it uses scientific and standardized methods to evaluate its benefits. When constructing a benefit factor evaluation system, it establishes an element system that includes the target layer, criterion layer, and scheme layer, and calculates the weights of each layer’s elements. The weight planning of sub criteria and project levels needs to comprehensively consider the importance, potential, and long-term impact on PH benefits of each factor. In the construction of the system, the relative weights between each factor are established by establishing a judgment matrix and utilizing expert ratings. After assignment, it is shown in Equation (1).

(1) $$\bar{Cnn}=\sqrt[{e}]{Cnn_{1}\times Cnn_{2}\times Cnn_{3}\times \cdots \times Cnn_{e}}$$

In Equation (1), $Cnn_{e}$ is the assignment made using the e -th expert’s suggestion, and $\bar{Cnn}$ is the geometric average of all assignments, which is the final position value (19, 20). Then, the weights of each factor can be determined by calculating the eigenvectors and maximum eigenvalues of the matrix, as shown in Equation (2).

(2) $$\left\{ \begin{array}{l} \overset{n}{\underset{i=1}{\sum }}Wi=1\\ CW=\lambda \max W\\ Wn=\bar{W}n/WP \end{array}\right.$$

In Equation (2), CW is the eigenvector of the matrix, λmax serves as the maximum eigenvalue of the matrix, and W serves as the normalized eigenvector. When the feature vector of the maximum eigenvalue becomes a weight vector for a certain degree of influence in the upper layer, if the inconsistency is significant, it will affect the accuracy of the judgment. Therefore, it is necessary to calculate consistency, as shown in Equation (3).

(3) $$CI=\left(\lambda \max-n\right)/\left(n-1\right)$$

In Equation (3), CI represents consistency. When the consistency reaches its optimal level, it is necessary to calculate the proportion, as showcased in Equation (4).

(4) CR=CI/RI

In Equation (4), *CR* serves as the consistency ratio and *RI* serves as the average random consistency indicator. When establishing a PH benefit evaluation system for smart cities, urban and regional differences should be considered. It first selects cities with moderate levels of PH services, optimizes projects on the ground of the number of service projects, and makes them reach the critical quantity to become residents’ health memory. Then, project quality screening is used to define projects that are easy to optimize and difficult to optimize, to improve project quality. It then sets a time range on the ground of the type of project. Finally, satisfaction indicators are set up to evaluate the subjective influencing feature factors (21, 22). This multi-level and multi angle evaluation system can provide a more comprehensive evaluation of PH benefits. The indicator system is shown in Equation (5).

(5) $$Di=\overset{n}{\underset{i=1}{\sum }}X_{i}P_{ij}$$

**Figure 2:**
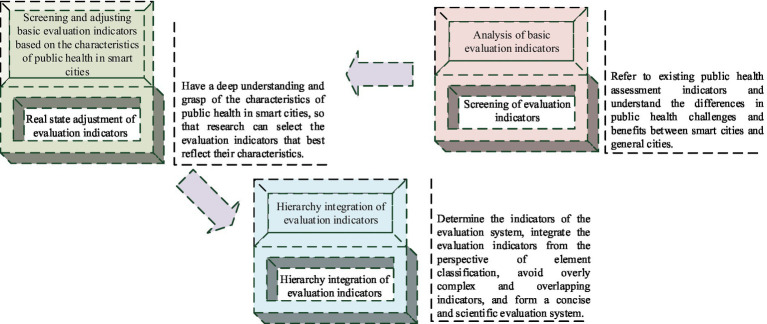
Construction of the evaluation system for PH benefit factors in smart cities.

In Equation (5), D serves as the indicator value, X is the evaluation value, and P serve as the statistical value of the indicator at different levels. To ensure the scientific and reasonable evaluation items in the evaluation system of PH benefits in smart cities, starting from the scientific construction of evaluation indicators, a construction method for the evaluation system of PH benefits in smart cities is established. This mainly includes three steps: screening evaluation indicators, adjusting the actual status of evaluation indicators, and integrating the hierarchy of evaluation indicators. On the ground of this, a smart city PH benefit factor evaluation system is constructed, as shown in Figure 2.

In Figure 2, when establishing a PH benefit evaluation system for smart cities, existing PH evaluation indicators should be used as a reference to understand the differences in PH challenges and benefit evaluations between smart cities and general cities. Then, on the ground of the characteristics of PH in smart cities, basic evaluation indicators are screened and adjusted. Then, it is differentiated and compared with the actual situation to determine the evaluation system indicators. Finally, it integrates evaluation indicators from the perspective of element classification, avoiding overly complex and overlapping indicators, and forming a concise and scientific evaluation system. According to the characteristics of PH in smart cities, it is necessary to screen and adjust basic evaluation indicators. When determining the indicators of the evaluation system, it is necessary to make a differentiated comparison with the actual situation. It integrates evaluation indicators from the perspective of element classification to avoid overly complex and overlapping indicators. The evaluation indicators can be divided into multiple aspects such as hardware facilities, software services, talent teams, and social environment. Each aspect has specific evaluation indicators, forming a concise and scientific evaluation system. Meanwhile, it is essential for adjusting and enhancing the evaluation system according to the actual situation to ensure its scientificity and effectiveness.

### 3.2 4 M smart city PH DNN model

**Figure 3:**
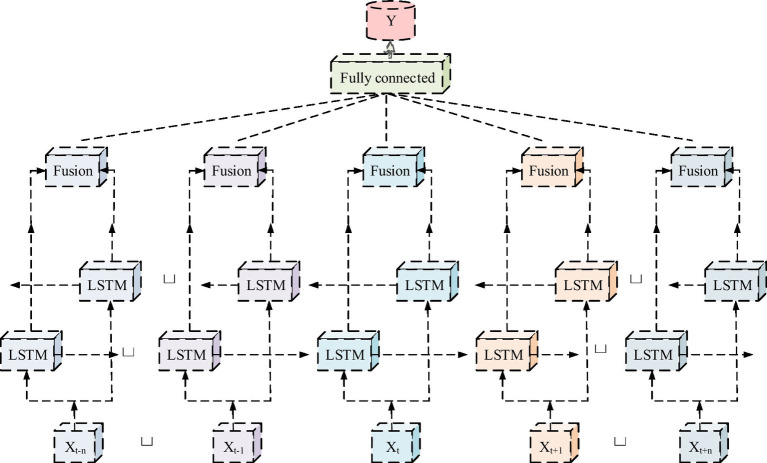
Structure of 4 M smart city PH Bi LSTM model.

On the basis of constructing an evaluation system for PH benefits in smart cities, a DNN model is presented for understanding the impact of factors on PH benefits. This model can extract valuable information from a large amount of data and reveal the performance of these factors in practical applications. The Bi LSTM model, as an upgraded version of LSTM, has unique advantages in extracting global information features (23, 24). It consists of two parts, namely standard LSTM and adding a layer of reverse LSTM. These two independent hidden layers jointly determine the final output value (OV) under the guidance of different parameters. The structure of the 4 M Smart City PH Bi LSTM model is shown in Figure 3.

In Figure 3, the Bi LSTM model still includes memory storage blocks, input gates, output gates, forget gates, and their related 8 parameters during the inference process. The goal of the model is to determine the most suitable values for these eight parameter factors through the training of the forward and reverse Bi LSTM structures. This is to capture the detailed features of historical and future information more comprehensively and flexibly, thereby achieving better prediction and performance improvement on the basis of high prediction accuracy of LSTM. The tanh function is an activation function (AF), known as the hyperbolic tangent function, utilized in the calculation of neuron state as well as output. Its formula is showcased in Equation (6).

(6) $$\tanh\left(x\right)=\frac{e^{x}-e^{-x}}{e^{x}+e^{-x}}$$

In Equation (6), the output of the tanh AF is located in the [−1, 1] interval, and the output is also 0 when the input is 0. Using tanh is equivalent to adjusting the input mean to 0. It helps with subsequent processing. When new information is received, $x_{t}$ and ${\tilde{C}}_{t}$ are multiplied and updated to the new cell state. The forget gate and input gate change the current cell state by probabilistic selection of the previous and current information. The process of updating the cell state from the original to the current state is ultimately remembered using Equation (7).

(7) $$C_{t}=C_{t-1}⊙f_{t}+i_{t}⊙{\tilde{C}}_{t}$$

In Equation (7), $C_{t-1}$ represents the cell state at time t−1 , and $f_{t}$ represents the forget gate state at time t . $C_{t}$ represents the new memory at time t , and $i_{t}$ serves as the input gate state at time t. The final memory $C_{t}$ of the current cell state is from the filtered information from the old cells and the new information. To apply the Bi LSTM network to the evaluation system of PH benefits in smart cities, the study integrates attention mechanism and Softmax function. The attention mechanism solves the problem of long-distance information dependence (25, 26). Its relevant formula is showcased in Equation (8).

(8) $$\alpha =\frac{\exp\left(e_{t,i}\right)}{\sum_{j=1}^{N}\exp\left(e_{t,j}\right)}$$

In Equation (8), $\exp\left(e_{t,i}\right)$ represents the OV of the Bi LSTM model. After passing through the attention mechanism layer to input PH information, the Softmax function is used to classify it. Its relevant formula is showcased in Equation (9).

(9) $$S\left(x_{j}\right)=\frac{e^{x}j}{\sum_{k=1}^{k}e^{x}k},j=1,2,\cdots ,K$$

In Equation (9), $S\left(x_{j}\right)$ represents the i-th dimensional value of the feature vector. k represents the quantity of categories. The formula about calculating classification labels is shown in Equation (10).

(10) $$\overset{⌢}{m}=\arg\max S\left(x_{j}\right)$$

In Equation (10), there is a very simple functional relation in the gradient of the cross entropy function and the OV of Softmax. This could get more excellent calculation. The loss function selects the classification cross entropy function, and its function is showcased in Equation (11).

(11) $$L=-\overset{T}{\underset{j=1}{\sum }}y_{j}\log s_{j}$$

**Figure 4:**
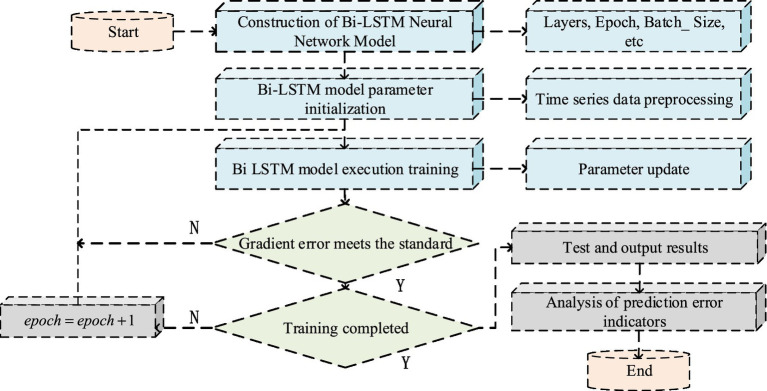
Execution process of 4 M smart city PH Bi LSTM model.

In Equation (11), $s_{j}$ represents the estimated probability. T serves as the quantity of categories classified (27, 28). The research aims to improve the accuracy of the Bi LSTM network by incorporating attention mechanisms and Softmax functions, providing better technical support for the development of PH in smart cities. The execution process of the Bi LSTM model is shown in Figure 4.

In Figure 4, from the perspective of Bi LSTM model training, the process is essential for establishing the model. The key is to continuously adjust and update the weights and related factor values to match the model and achieve more accurate prediction results. This model analyzes past and future data and can explore data characteristics in detail, reducing the impact of other interference factors, thus enabling more accurate speed prediction than LSTM. To prevent overfitting, a Dropout layer can be added, as showcased in Equation (12).

(12) $$X_{i}^{\left(t+1\right)}=\mathrm{dropout}\left(h_{i}^{\left(t\right)},p\right)$$

In Equation (12), X is the input, h is the output, p serves as the Ddropout rate, and N serves as the quantity of neuron layers.

### 3.3 A deep model of PH benefits in smart cities on the ground of DNNs from a 4 M perspective

**Figure 5:**
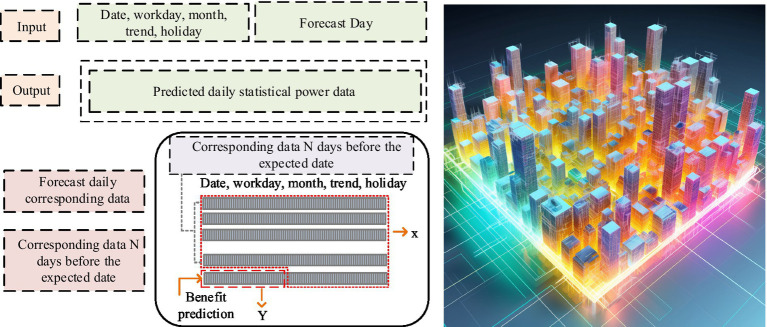
Deep model of PH benefits in smart cities.

The previous section provided a detailed introduction to the Bi LSTM based DNN model and its utilization in the evaluation of PH benefits in smart cities. This section explores the construction of a deep model for PH benefits in smart cities on the ground of DNNs. This is to provide a more comprehensive and in-depth tool for evaluating PH benefits. The deep model of PH benefits in smart cities adopts a DNN model with three-dimensional data input, as shown in Figure 5.

In Figure 5, on the ground of the data from historical PH benefit sampling points, the study can make detailed predictions of different external factors such as dates, working days, months, trends, holidays, etc., and provide corresponding outputs for each sampling point. When setting up the network structure, the hidden layer can be a separate layer or multiple layers. Single hidden layer networks perform well in handling complex problems, while multi hidden layer networks can provide higher prediction accuracy. However, as the quantity of hidden layers increases, the network structure becomes more complex and the training time is correspondingly extended. The experience hiding layer nodes are shown in Equation (13).

(13) $$m=\sqrt{l+n}+\alpha$$

**Figure 6:**
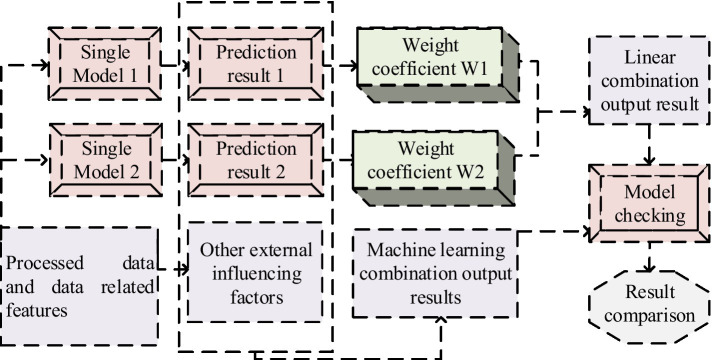
Integrated learning prediction model framework.

In Equation (13), m serves as the quantity of hidden layer nodes, l serves as the quantity of input layer nodes, n serves as the quantity of output layer nodes, and α is a constant from 1 to 10 (29, 30). Neural networks use AFs to incorporate nonlinear factors into the model. Due to the positive historical data and predicted results of PH benefits, the AF from the input layer to the hidden layer of the neural network will use the ReLU function. The AF from the hidden layer to the output layer will use the Linear function. The ensemble learning prediction model is used to improve model accuracy, and its process framework is shown in Figure 6.

In Figure 6, the forecast data of two or more individual models are mainly integrated to obtain a comprehensive forecast result. By analyzing a single model, it selects a model with good predictive performance and overlays it. The combination model on the ground of the average weight method is the most convenient. It directly assigns the same weight to a single model without considering any situation, as shown in Meanwhile (Equation 14).

(14) $$f_{\mathrm{prei}}=\frac{1}{q}\left(i=1,2,\ldots ,q\right)$$

In Equation (14), q represents the model, i serves as the weight of the i -th model, and is further extended to the formula, as showcased in Equation (15).

(15) $$f_{pre}=\overset{n}{\underset{i=1}{\sum }}y_{i}f_{\mathrm{prei}}$$

In Equation (15), $f_{pre}$ is the prediction result of the ensemble method, and $f_{\mathrm{prei}}$ is the prediction result of the i -th model. This method is an innovative technique that utilizes polynomial joint modeling by weighting individual patterns with small deviations to reduce joint modeling errors and improve prediction accuracy. This method not only considers the interaction between different modes, but also accurately corrects the small deviations of each mode, making the forecast results more accurate and reliable. In the specific implementation process, polynomial fitting is performed on each mode to obtain the polynomial coefficients of each mode. Then, on the ground of the polynomial coefficients of each pattern, it calculates the weighted coefficients of each pattern. It then multiplies the weighted coefficients of each pattern with the corresponding polynomial coefficients to obtain the polynomial coefficients for joint modeling, thereby obtaining the final forecast result. The error used in the combined model is the average absolute percentage error, and the formula for the integrated model using the reciprocal error method is shown in Equation (16).

(16) $$\left\{ \begin{array}{l} \omega_{1}=\frac{\varepsilon_{2}}{\varepsilon_{1}+\varepsilon_{2}}\\ \omega_{2}=\frac{\varepsilon_{1}}{\varepsilon_{1}+\varepsilon_{2}}\\ f_{pre}=\omega_{1}f_{pre1}+\omega_{2}f_{pre2} \end{array}\right.$$

In Equation (16), $\omega_{i}$ serves as the weight coefficient, and $\varepsilon_{i}$ serves as the error of each model.

## 4 Deep application effects of PH challenges and benefits in smart cities on the ground of the 4 M perspective

Exploring the challenges and benefits of PH in smart cities from a 4 M perspective, focusing on the evaluation and performance comparison of deep application effects. This is to evaluate the in-depth effectiveness of smart city PH applications on the ground of the 4 M perspective, to understand the impact of different factors on PH benefits. Further comparison of the in-depth performance of the research aims to reveal the advantages and challenges from different perspectives, providing reference for improving the PH benefits of smart cities.

### 4.1 Deep application effects of PH challenges and benefits in smart cities on the ground of the 4 M perspective

In the process of exploring the challenges and benefits of PH in smart cities, analyzing the characteristics of the 4 M perspective is an indispensable part. The characteristics of this aspect have a profound impact on the effectiveness of smart cities in PH challenges. The experimental parameters are set as shown in Table 1.

**Table 1:** Subjective mood survey results.

Parameter	Value/Model	Illustrate
Processor	Intel Core i7	Computing hardware for running deep learning models
Memory	16GB DDR4	Hardware for storing intermediate operation results
Hard disk	1 TB SSD	Hardware for storing data and models
Operating system	Windows 10	Software environment for running the model
Programming language	Python 3.7	Software tools for writing and running models
Deep learning framework	TensorFlow 2.0	The main software library for implementing the Bi LSTM model

**Figure 7:**
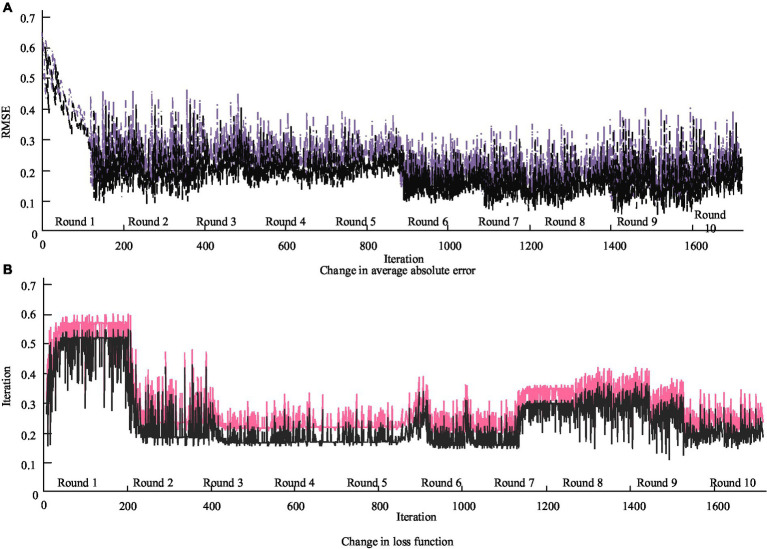
Changes in cross entropy loss values of deep application effects of PH in smart cities from a 4 M perspective. **(A)** Change in average absolute error. **(B)** Change in loss function.

It delves into the changes in cross entropy loss values of the deep application effect of PH in smart cities on the ground of the 4 M perspective, as shown in Figure 7.

**Figure 8:**
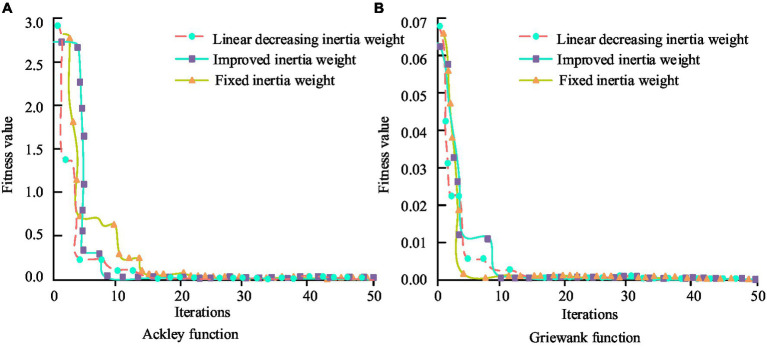
Iterative convergence curve of deep application of PH in smart cities under different model algorithms. **(A)** Ackley function. **(B)** Griewank function.

In Figure 7, the trend sequence of the training set gradually decreases. By the third round of training, the loss function has basically stabilized, and by the tenth round, the function no longer decreases. This phenomenon indirectly proves that the model can fit normally and achieve optimal accuracy. Different amounts of recent historical data will have different impacts on the prediction results. The iterative convergence curves of deep application of PH in smart cities under different model algorithms are shown in Figure 8.

**Figure 9:**
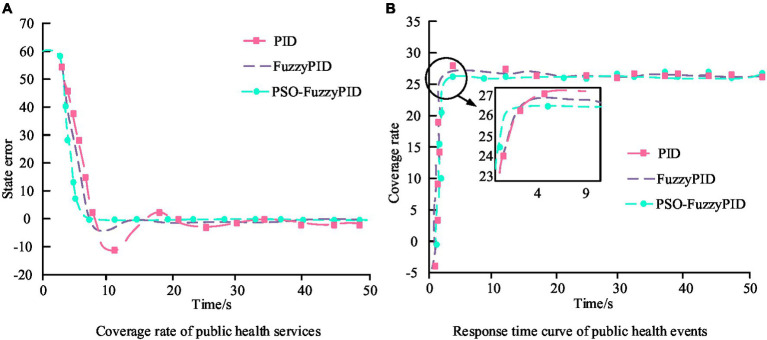
Deviation correction curves for PH challenge applications under different model algorithms. **(A)** Coverage rate of public health services. **(B)** Response time curve of public health events.

In Figure 8, the improved dynamic inertia weight algorithm converges slowly in the initial stage and has some distance from the global optimal fitness value. However, compared with linearly decreasing inertia weights and fixed inertia weights, the improved inertia weight Ackley function converges at the 25th and 14th iterations. The convergence speed is three times and two times faster than the other two algorithms. In terms of the Griewank function, the improved inertia weight falls into local optimal fitness values during the second and sixth iterations. From the perspective of iterative convergence efficiency and avoiding getting stuck in local optima, the improved dynamic inertia weight algorithm is significantly superior to the other two algorithms. In the in-depth application research of PH in smart cities, the Bi LSTM model is widely adopted. It optimizes the model on the ground of an improved Particle Swarm Optimization (PSO) algorithm. The purpose is to reduce the overshoot, steady-state error, adjustment time, and response time of the deviation curve between PH service coverage and PH event response time. The bias correction curves for PH challenge applications under different model algorithms are shown in Figure 9.

**Figure 10:**
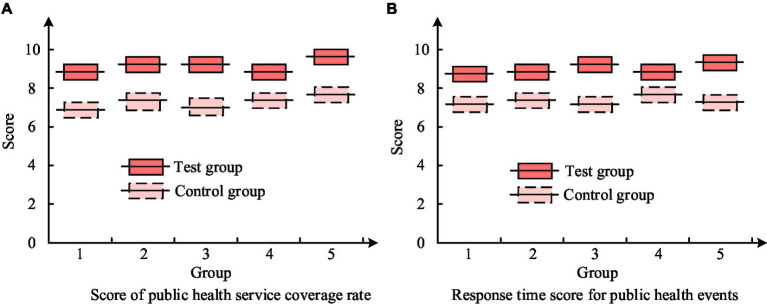
Results of PH service coverage and response time for two sets of samples. **(A)** Score of public health service coverage rate. **(B)** Response time score for public health events.

Figure 9 is on the ground of MATLAB software. The results showed that the coverage rate of PH services and the overshoot of the PH event response time curve were reduced by 96.08 and 89.48%, respectively, compared to ordinary PID. The steady-state error was reduced by 1.24%, the response time was shortened by 12.4 and 59.98%, and the adjustment time was shortened by 86.37 and 80.01%, respectively. In terms of resource allocation and process optimization, through reasonable adjustments and optimizations, the coverage rate of PH services has increased by 20%. Meanwhile, the response time to PH incidents has been reduced by 30%. In the actual application effect testing of the evaluation model, comparative analysis method is used to select samples with the same basic information for comparison. The experimental group (EG) adopted PH services integrated with improved PSO optimization models, while the control group (CG) adopted routine PH services. The results of PH service coverage and response time for two sets of samples are shown in Figure 10.

Figure 10A shows that the PH service coverage of the EG is higher than that of the CG among the five samples. And the average PH service coverage rate of the EG was 8.9 points, exceeding the CG’s 7.3 points. Figure 10B shows that among the five samples, the EG scored higher in response time to PH events than the CG. And the average PH event response time score of the EG was 8.7 points, exceeding the 7.5 points of the CG.

### 4.2 Comparison of deep performance of PH challenges and benefits in smart cities from a 4 M perspective

**Figure 11:**
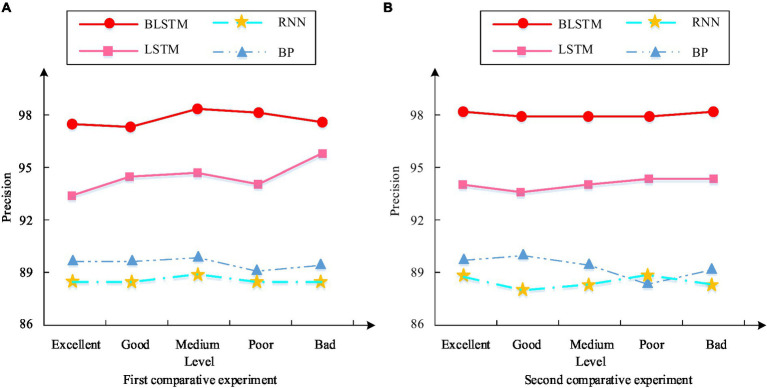
Accuracy results of PH benefits in different deep learning models. **(A)** First comparative experiment. **(B)** Second comparative experiment.

After in-depth analysis of the challenges and benefits of PH in smart cities on the ground of the 4 M perspective, the influence and role of various perspectives in PH management have been clarified in this study. Further explore performance issues to empirically verify the actual effectiveness of the model in PH applications. It conducts in-depth comparisons from multiple dimensions such as precision, recall, and F1 value. And it analyzes the improvement of PH service efficiency in smart cities from the perspective of 4 M, to provide more accurate reference basis for PH management in smart cities. The accuracy results of PH benefits in different deep learning models are shown in Figure 11.

**Figure 12:**
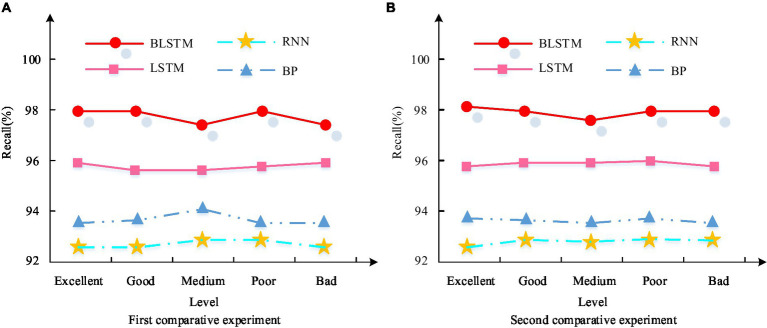
Recall rate results of PH benefits in different deep learning models. **(A)** First comparative experiment. **(B)** Second comparative experiment.

Figure 11A showcases the results of the first comparative experiment. This indicates that the average accuracy of the BiLSTM model in five different categories of datasets is 98.6%, significantly higher than the other three models. This result indicates that the BiLSTM model can provide high predictive accuracy and effective evaluation support for PH benefits when dealing with PH challenges in smart cities. Figure 11B reflects the results of the second comparative experiment. This indicates that the average accuracy of the BiLSTM model in five different categories of datasets has been further improved to 89.3%, still better than the other three models. This result further confirms the robustness and superiority of the BiLSTM model in the study of deep application effects in PH. The recall results of PH benefits in different deep learning models are shown in Figure 12.

**Figure 13:**
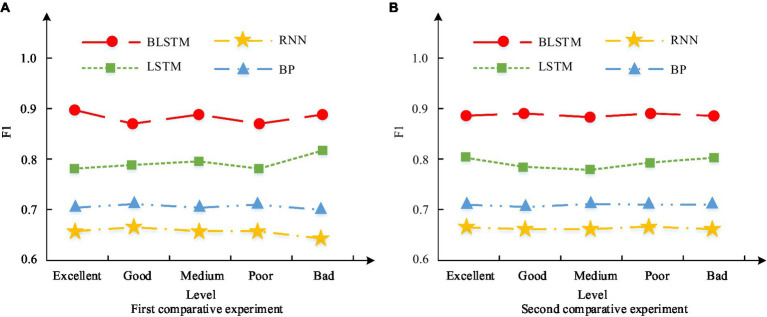
F1 value results of PH benefits in different deep learning models. **(A)** First comparative experiment. **(B)** Second comparative experiment.

In the first, the recall curve of the BiLSTM model was the best. And the average recall rate in five different categories of datasets reached 98.2%, which is higher than the LSTM model’s 95.7%, BP model’s 95.1%, and RNN model’s 93.11%. In the second comparative experiment, as shown in Figure 12B, the recall curve of the BiLSTM model still exceeds others. And its average recall rate in five of datasets further increased to 98.2%, higher than the 96.8% of LSTM, 94.9% of BP, and 93.7% of RNN. The F1 value results of PH benefits in different deep learning models are shown in Figure 13.

**Figure 14:**
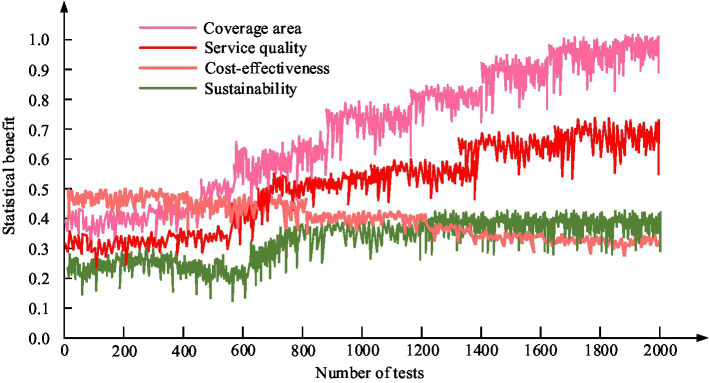
Efficiency results of Smart City PH service application based on 4 M perspective.

In the first comparative experiment, as shown in Figure 13A, the F1 value curve of the BiLSTM model exceeded others. And its average F1 value in five of datasets is 0.88. It exceeds the LSTM model’s 0.79, BP model’s 0.70, and RNN model’s 0.65. In the second comparative experiment, as shown in Figure 13B, the F1 value curve of the BiLSTM model still exceeds others. Its average F1 value in five of datasets further increased to 0.89, higher than the LSTM model’s 0.78, BP model’s 0.71, and RNN model’s 0.66. The efficiency results of the application of PH services in smart cities from the perspective of 4 M are shown in Figure 14.

In Figure 14, the service quality and coverage range increased from 0.32 to 0.62 and 0.41 to 0.93, respectively, reflecting a significant improvement in service quality and coverage breadth. However, the decrease in cost-effectiveness from 0.42 to 0.33 suggests that further optimization of economic benefits may be needed. The increase in sustainability from 0.23 to 0.31 demonstrates the long-term effectiveness of services and their ability to adapt to environmental changes.

## 5 Discussion and implications

Research provides important insights into public health management for smart cities; However, they need to be discussed more strongly in the context of existing literature and the research questions initially proposed. Although this study successfully demonstrated the potential of innovative PH management from a 4 M perspective - combining humans, machines, materials, and methods - there are gaps in directly addressing the consistency or differences between these findings and previous research. For example, the integration of DNN for predicting health issues has been explored before, but the improvement of these predictions and their practical significance in smart cities have not been fully compared with existing models in this study. The theoretical basis for how the four components studied interact in the pH background has not been widely elaborated. Future research should develop a more detailed theoretical model that describes the interactions between humans, machines, materials, and methods. In terms of policy, this study indirectly supports the adoption of advanced technologies and cross departmental collaboration in PH management. However, there is a lack of specific policy recommendations. Policy makers can benefit from the guidance of implementing the 4 M framework in different government scales and urban backgrounds. When considering practice, the operation of this framework in daily PH management is still abstract for practitioners. It is necessary to clearly explain how PH professionals apply the results of this study, possibly through case studies or best practice guidelines. Finally, the limitations of this study have not been thoroughly examined. For example, the scalability of the 4 M framework and the proposed technological solutions may face significant obstacles in low resource environments or different cultural backgrounds.

## 6 Conclusion

The development of smart cities and the acceleration of urbanization have brought new challenges to PH management, especially how to quickly and accurately respond to large-scale PH events. To address this challenge, the study takes a 4 M perspective and explores the depth of PH management issues. It introduces a machine perspective and constructs a PH prediction model on the ground of DNNs. The research results indicate that the PH benefits of smart cities from a 4 M perspective are significant. Specifically, the overshoot of PH service coverage and PH event response time decreased by 96.08 and 89.48%, respectively, compared to ordinary PID, with a steady-state error reduction of 1.24%, response time shortened by 12.4 and 59.98% respectively, and adjustment time shortened by 86.37 and 80.01%, respectively. In addition, the PH service coverage rate of the EG exceeded that of the CG, with an average score of 8.9, exceeding the CG’s 7.3. On the ground of DNN prediction models, the accuracy and F1 value of the BiLSTM model are significantly higher than other models. The contribution lies in introducing the 4 M concept into PH management and combining DNN technology to provide new theoretical tools for PH prediction and management. However, there are still some shortcomings in the research, such as the need for more in-depth segmentation of groups from a human perspective. In the machine perspective, DNNs need further optimization. From the perspective of materials and methods, further in-depth research is needed. Looking ahead to the future, it is expected that research methods on the ground of the 4 M perspective can be promoted and applied globally. This can contribute to the PH management of smart cities and help them respond to PH events faster and more accurately. Implementing the proposed research plan is expected to enhance public health services in smart cities by offering personalized healthcare interventions, predicting outbreaks using data analytics, and optimizing resource allocation. For large-scale public health events, this approach could enable rapid, data-driven responses, ensuring timely and targeted measures are taken to mitigate risks and manage situations effectively, thereby improving overall public health resilience.

## Data availability statement

The original contributions presented in the study are included in the article/supplementary material, further inquiries can be directed to the corresponding author.

## Author contributions

LY: Investigation, Writing – original draft. LD: Investigation, Supervision, Writing – review & editing. YG: Investigation, Writing – original draft. YZ: Methodology, Writing – review & editing. YS: Conceptualization, Writing – review & editing.
